# Male Courtship Behavior of the South American Fruit Fly, *Anastrepha fraterculus*, from an Argentinean Laboratory Strain

**DOI:** 10.1673/031.011.17501

**Published:** 2011-12-31

**Authors:** P. Gomez Cendra, G. Calcagno, L. Belluscio, J.C. Vilardi

**Affiliations:** Laboratorio de Genética de Poblaciones Aplicada, Facultad de Ciencias Exactas y Naturales (FCEN), Universidad de Buenos Aires, Buenos Aires, Argentina

**Keywords:** mating, sterile insect technique, tephritids

## Abstract

The South American fruit fly *Anastrepha fraterculus* (Wiedemann) (Diptera: Tephritidae) is a pest of fruit species of warm regions of the Americas, including Argentina. Some authors claim that this taxon includes a group of cryptic species. In order to evaluate possible targets of sexual selection, it is necessary to analyze ethological aspects of male courtship and identify particular steps that strongly influence mating success. A mating test designed to evaluate behavioral differences between insects that achieve copulation (successful males) and those that did not mate (unsuccessful males) could also be relevant for the possible implementation of control programs based on sterile insect technique. Reared insects need to be evaluated periodically, since genetic drift and artificial selection associated with rearing conditions could have a detrimental effect on their ability to compete for matings in nature. In this study, courtship behavior of *A. fraterculus *males from a laboratory strain was analyzed for the first time through video recordings. Three components for the activities were identified: calling, wing positions, and movements. Also, the time that males spent on each step of the courtship was registered, including the last activities before attempting copulation. Data showed that mating achievement occurs relatively quickly; 65% of the successful males reached copulation within the first ten minutes after the male and female were placed together. Behavioral differences were detected between successful and unsuccessful males. The former group tended to invest more time in activities directly related with mating (Spin, Arrowhead, Attempt); however, as courtship progressed, unsuccessful males increased the time dedicated to activities not directly associated to mating (Call 0, Relax,Stationary). There was not a single sequence of activities leading to success, but the analysis of the last activities performed before mating attempts indicated that the most frequent position before successful attempts was Arrowhead, occurring in 68% of cases, whereas in unsuccessful males this position was observed only 18% of the time before mounting. Although the behavior of the strain analyzed here should be compared with that of natural populations, one would not expect to observe significant differences as compatibility and competitiveness with wild collected flies was previously shown under field cage conditions. Behavioral tests such as those applied here might be important to assess quality of mass reared strains for sterile insect technique implementation programs.

## Introduction

The South American fruit fly *Anastrepha fraterculus* (Wiedemann) (Diptera: Tephritidae) is distributed in tropical and subtropical regions of the Americas. In Argentina this species is abundant in the northeast and northwest regions (Vergani 1956). These areas are characterized by hot and wet subtropical climate and are separated by an arid central area (Cabrera and Willink 1980). *Anastrepha fraterculus* uses riping fruits as ovipositing sites, and larvae produce severe damage to commercially important fruits (Malavasi et al. 2000; Ovruski et al. 2003).

Wide variation has been observed among populations of *A. fraterculus* from different regions in the Americas, as reflected by morphological studies and biochemical, genetic, and molecular markers (Stone 1942; Baker et al. 1944; Morgante et al. 1980; Solferini and Morgante 1987; Steck and Sheppard 1993; Selivon et al. 1997, 1999, 2001, 2005), supporting the hypothesis that *A. fraterculus* is a complex rather than a single biological species.

Relevant information to distinguish synmorphic species can be obtained from the analysis of reproductive isolation mediated by differences in courtship behavior (ethological isolation) (Dobzhansky 1937). In sexual reproductive organisms, reproductive isolation can be considered as the critical step in the process of speciation (Mayr 1963). An alternative definition of “biological species” is based on the specific recognition patterns required for mating (Paterson 1978, 1985), which also considers sexual behavior as a crucial condition to define a species.

In order to discriminate biological species within the *A. fraterculus* complex, it is important to directly test the mating compatibility among different populations. Mating compatibility tests gave no evidence of isolation among Argentinean populations of *A. fraterculus* (Petit-Marty et al. 2004), but populations from different countries showed variable degrees of behavioral isolation (Vera et al. 2006). These results are consistent with molecular findings (Alberti et al. 2002, 2008). It would be particularly interesting to analyze the activities displayed during male courtship and identify those steps that determine mating success.

Mate choice is a central evolutionary process, since it is a main component of sexual selection (Heisler et al. 1987). There is a wide range of species, including tephritids, where females choose a mating partner from among several displaying males. This kind of sexual selection (intersexual) favors the evolution of elaborate displays by courting males, as females can actively resist mating attempts and only allow copulation after being “convinced” by the male behavior (Holland and Rice 1998; Gavrilets et al. 2001; Kokko et al. 2003). Many tephritids exhibit a lek mating system (Shelly and Whittier 1997; Aluja et al. 2000; Shelly 2001) in which males aggregate and release pheromones to attract females for the sole purpose of mating (Bradbury 1981; Shelly and Whittier 1997). *Anastrepha fraterculus* lek formation has been analyzed in Brazil by Malavasi et al. (1983) and in Argentina by Segura et al. (2007). Female selection criteria may be not obvious and may take place both before and after copulation.

The comparison between the behavior of wild and laboratory-reared insects is relevant to the implementation of the sterile insect technique. This method (Knipling 1959, 1968) involves massive rearing and liberation of insects sterilized by gamma irradiation in order to compete for matings with wild insects in the field. These matings are expected to leave no offspring (Cunningham et al. 1980; Klassen et al. 1994). In Argentina, sterile insect technique is being successfully implemented to control another tephritid, *Ceratitis capitata *(Aruani et al. 1996; De Longo et al. 2000). Efficiency of this kind of control programs is dependent on routine monitoring of the quality of the laboratory reared strain, mainly in reference to survival and mating competitiveness in the field, because genetic drift and artificial selection may have a detrimental effect on them (Leppla 1989; Cayol 2000). Even in cases where laboratory strains were originally fully compatible with wild flies, adaptation to artificial rearing for many generations may result in behavioral changes, including courtship activities, which must be monitored in order to avoid a reduction in competitiveness (Shelly et al. 1994; Lance et al. 2000; Alphey 2002; Benedict and Robinson 2003).

In the wild, *A. fraterculus* males congregate and release pheromones early in the morning (Petit-Marty et al. 2004; Segura et al. 2007) as part of a lek behavior destined first to attract the female and then to achieve copulation, usually referred to as calling. When a female approaches the lek, males display several courtship activities with the purpose of being chosen by the female as the mating partner. A preliminary study by Calcagno and Vilardi (2001) recorded different steps of the *A. fraterculus* courtship and identified two groups of activities in reference to the distance between male and female: 1) long distance and 2) close up activities. The first group included male calling and wing fanning. During calling, males expand the pleural abdominal region producing two lateral blisters while holding a pheromone drop surrounded by rectal epithelium in the abdomen end. Wing fanning involves continuous wing vibrations. The close up activities occur when the male and female face each other and several interactions occur, including wing signaling, mating attempts (mounting), and even fights when the female is not receptive. Other studies have shown that wing beats and/or typical rightward and leftward circular movements are frequent, probably to enhance the pheromone dispersal (Arita and Kaneshiro 1989; Briceño and Eberhard 2002). More precise observations are needed to achieve a better knowledge of courtship behavior.

The main objectives of this paper were: 1) to typify the male courtship intended to promote female acceptance through the identification of its most frequent behaviors and the recording of the time dedicated to each activity; 2) to detect, when possible, behaviors that are directly related with mating success; and 3) to increase the general knowledge about *A. fraterculus*, and to generate relevant data that could be useful for the implementation of sterile insect technique in the control of *A. fraterculus* in Argentina.

## Materials and Methods

### Biological material

Analyzed insects were obtained from a laboratory strain established in 1997 with a semi-massive management system (Jaldo et al. 2001; Vera et al. 2007) at the Estación Experimental Agroindustrial Obispo Colombres, Tucumán Province, Argentina. This strain originated from a wild population from an uncultivated guava orchard in the same region. In June 2004, pupae were sent to the Instituto de Genética, Instituto Nacional de Tecnología Agropecuaria, Castelar, Buenos Aires Province, Argentina where they were maintained in a rearing room with controlled conditions of 23.5 ± 1 ^°^C, 70 ± 10% RH, and 12:12 L:D until adult emergence. There were four cohorts. Adults were assorted by sex 48 or 72 hours after emergence in order to assure their virginity, and placed in 3 L glass flasks (about 90 rearing flasks were prepared) containing no more than 40 individuals. They were provided with *ad libitum* water and solid diet based on brown sugar and hydrolyzed maize protein (Manso 1998). This particular diet has been shown to enhance the normal sexual development in laboratory (Manso 1998). Under these conditions flies reached sexual maturity in 12 days on average.

#### Data collection

Adults were video recorded according to the protocol used by Calcagno et al. (2002) to describe *C. capitata* mating behavior, with some modifications related to recording times and lightning conditions.

Around 08:30 each day, one randomly chosen sexually mature male (16 ± 2 days old) was placed in each of five transparent acrylic cylindrical cages (7 cm tall, 8.5 cm diameter) through a small lateral hole. Starting time was chosen to match the mating peak period for this species in Argentinean populations (Petit Marty et al. 2004). Suitable illumination was attained by conducting the experiment beside a large window to obtain natural daylight. Temperature ranged from 21–26 ^°^C and RH from 54–72%. Inside the cage and on the top, a lemon (*Citrus limon*) leaf was fixed with tape to mimic natural conditions; most matings in the wild take place in the abaxial leaf side. When one of the five males began calling with visible releasing of pheromone, a Sony Hi 8 CCD-TR805 video camera (www.sony.com) with a Novoflex macro lens (www.novoflex.com) was placed under the cage. The camera was wired to a JVC H-J401EN model video recorder (www.jvc.com) and a Philips 14GX1510/77B color television (www.philips.com). The fly was recorded for 10 min, verifying the recording quality. Afterward, without stopping the recording, a female (same age) was released inside the cage, and male activities were recorded for an additional 30-minute period. If copulation occurred, starting and ending times were scored, even if the couple finished after the recording time. 48 video recordings were obtained. Seven were used as pilot tests to improve the videotaping procedure, and the remaining 41 courtships were fully analyzed.

#### Video observation

All videos were carefully observed, and male behaviors were identified and characterized in detail by means of the frame-by-frame function of the video recorder. Duration of each behavioral activity was measured with the video recorder counter. Therefore, a complete record of all male activities displayed second-by-second was obtained, and all the general behavior and the particular movements at each given instant were characterized. The amount of time dedicated to each activity was registered, including any kind of physical interaction between male and female and the couple's activity while mating.

#### Data analysis

Males that copulated within the 30-min recording period were considered “successful”, and the remaining males were labeled as “unsuccessful”. The average number of copulation attempts was compared between these groups applying a generalized lineal model, assuming that the number of attempts follows a Poisson distribution. The time spent on each activity before the female was placed on the cage was compared between successful and unsuccessful males by MANOVA and individual ANOVAs. To simplify the analysis, three MANOVAs were conducted, one for each component of activities as described below (see Table 1). The overall significance of the MANOVA was evaluated by Wilks' lambda test. When possible, a Spearman's rank correlation test was also performed to determine if the relative proportion of time dedicated to each activity was similar in successful and unsuccessful males.

Total courtship duration (*T*) was defined as the elapsed time from the moment the female was placed into the cage with the male to the moment when copulation started or the end of the recording period (30 min). The regression of the proportion of time dedicated to each activity (dependent variable) on courtship time (explanatory variable) was analyzed. To make the observations comparable, the explanatory variable was expressed as the ratio (t_r_),


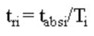


where t_absi_ is the absolute time (in seconds) elapsed from female release, and Ti is total courtship duration for male i. This transformation was necessary due to the fact that total courtship duration was variable from individual to individual depending on the outcome of courtship, ranging from a few seconds to 30 minutes, the maximum observation period (for unsuccessful males). In order to evaluate the behavioral differences between successful and unsuccessful males, an analysis of covariance (ANCOVA) for each activity was conducted, which included success as a covariate in the regression model.

To further test behavioral differences that may be directly related to mating achievement, the time invested by each individual on each activity during the five seconds previous to mating attempts was analyzed in detail. These data were focused on three groups and the activities before (a) failed attempts by unsuccessful males, (b) failed attempts by individuals that obtained copulation in later attempts, and (c) attempts leading to copulation. It should be noted that groups (b) and (c) correspond to attempts by successful individuals. Differences between the three groups were evaluated by means of contingency tables, and the significance was estimated by Monte Carlo tests (2000 permutations). Differences of frequencies of the last recorded activity before each attempt were also compared among the three groups described above by contingency tables and Monte Carlo tests. All analyses were conducted with the R stats package (version 2.11.1) (R Development Core Team 2010).

## Results

Of the 41 males recorded in this study, 31 (75%) mated within the 30 min of recording and were considered successful (S), whereas the remaining 10 males were considered unsuccessful (U). Nearly half of successful males (14/31) reached copulation within the first five minutes after the female was releasedinto the cage. Observation of the video allowed the identification of male activities and the elimination of some as being very frequent but apparently unrelated with courtship (i.e., wing cleaning). The complete list of activities is described in Table 1. Each activity was described using three components: (a) calling by pheromone release, (b) wing position, and (c) movement. With different states for each component— three for calling, eight for wing position, and six for movement—there were a total of 144 possible unique activities. Such a large number of possibilities involves statistical restrictions as many classes are empty or represented by only a few cases. In order to simplify the analysis, each component was analyzed separately.

**Table 1.**
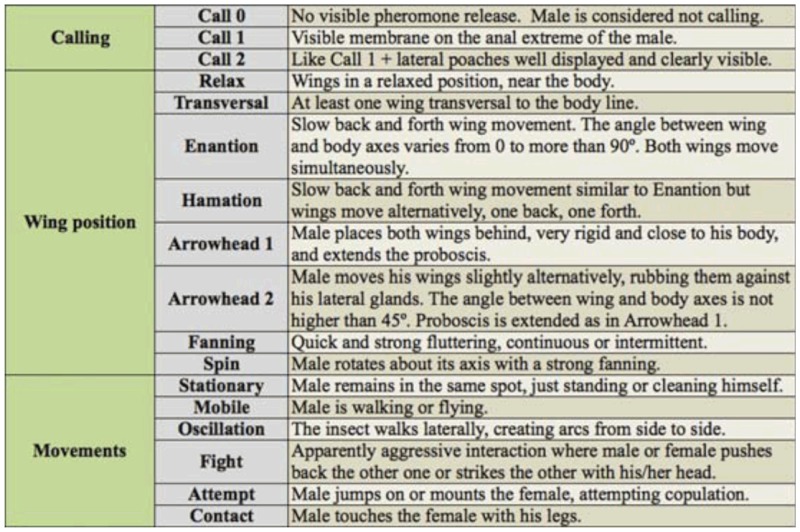
List of identified courtship activities displayed by *Anastrepha fraterculus* males.

Terminology for the behavioral activities is based mainly on earlier work on *C. capitata *(see Calcagno et al. 2002) and other fruit flies (White et al. 2000). ‘Arrowhead’ is a term frequently used in analysis of the genus *Anastrepha* (Dodson 1982), but here a distinction was made between two slightly different phases referred to as ‘arrowhead 1’, with motionless wings, and ‘arrowhead 2’, when the male gently moves his wings alternatively backwards and forwards. In a similar way, three call types were distinguished, according with the visibility of the anal membrane and lateral poaches. ‘Enantion’ (slow back and forth movements with both wings at the same time) and ‘hamation’ (slow back and forth movements alternating the two wings) were taken from Robacker and Hart (1985), and here the term ‘oscillation’ is introduced for the male walking in an arc-shaped pattern. Behavior analysis of activities displayed in female absence or female presence were conducted separately.

**Table 2.**
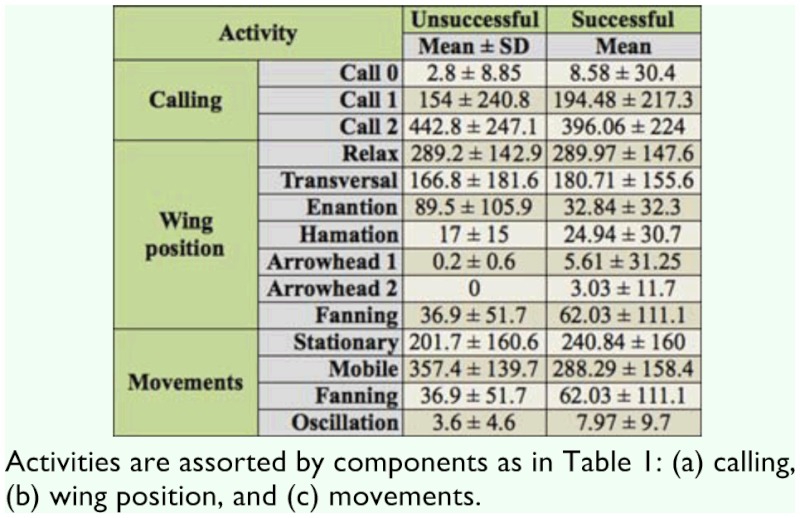
Mean and standard deviations for time spent in each activity (in seconds) by successful (S) and unsuccessful (U) males of *Anastrepha fraterculus* during the 10 min of video recording of the male alone (before female release inside the cage).

### Activities during the 10 minutes before female release

The average duration of each activity displayed by the male was measured for S and U individuals. For calling activities, the comparison of the basic statistics of the time dedicated to each activity (see Table 2a) by MANOVA (Wilks' lambda = 0.99, *p* = 0.91) and individual ANOVAs (*F* = 0.25–0.35, *p* = 0.56–0.62) did not show any significant difference between S and U males.

In reference to wing positions (Table 2b), according to the individual ANOVAs, successful males allocated significantly less time to Enantion than unsuccessful ones (*F* = 7.16, *p* < 0.01), although the MANOVA failed to show overall significant differences between groups (Wilks' lambda = 0.74, *p* = 0.146). The times spent on each wing activity were highly significantly correlated between S and U individuals (Spearman's rho = 0.96, *p* < 0.01).

Finally, the comparison of movements displayed by males (Table 2c) showed no significant differences between groups in any case (Wilks' lambda = 0.90, *p* = 0.42; individual ANOVAs *F* = 0.45–1.85, *p* = 0.18– 0.51). The correlation of movements between S and U males was high (Spearman's rho = 1), but not significant (*p* = 0.083), probably due to the low number of activities considered (four).

**Table 3.**
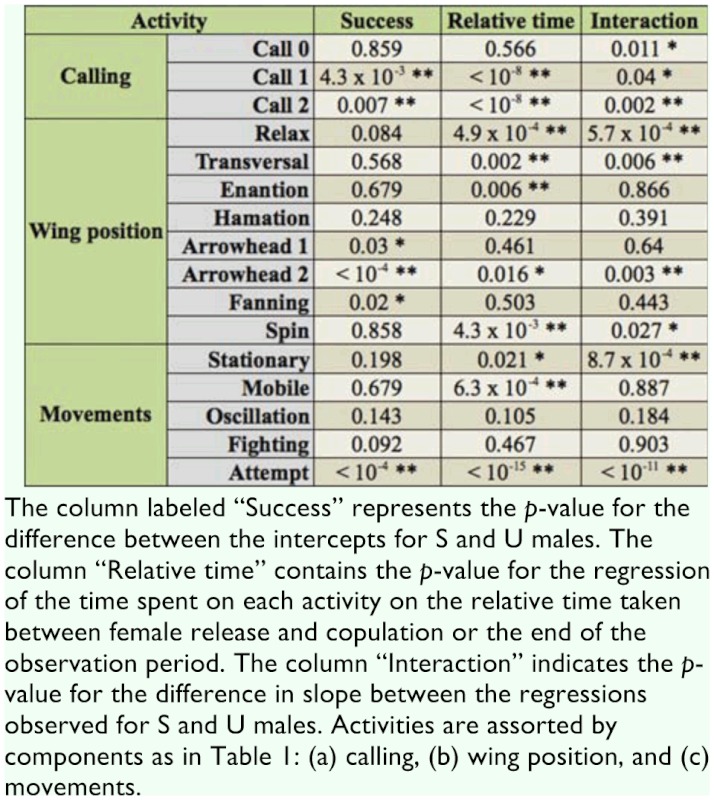
Results of the analysis of covariance (ANCOVA) of the time spent in each activity on courtship time by (S) and (U) males of *Anastrepha fraterculus* during the 30 min of video recording with the female inside the cage.

### Activities displayed by males after female release

During the trial, males spent more than 90% of the time on releasing attractant pheromone (Call 1 and 2). Thus, it appears that this activity can be sustained at least as long as the whole experimental period (40 minutes of our total video recording time)

Males (S and U pooled) spent nearly 40% of the courtship time in a quiet position, with the wings towards the posterior extreme of the body (Relax). They were Stationary, on average, 37% of the time, but the most common activity (Mobile), taking ∼ 50% of the time, was walking or flying. Only a quite small fraction of the time (∼ 4%) was spent in Attempts.

**Figure 1.**
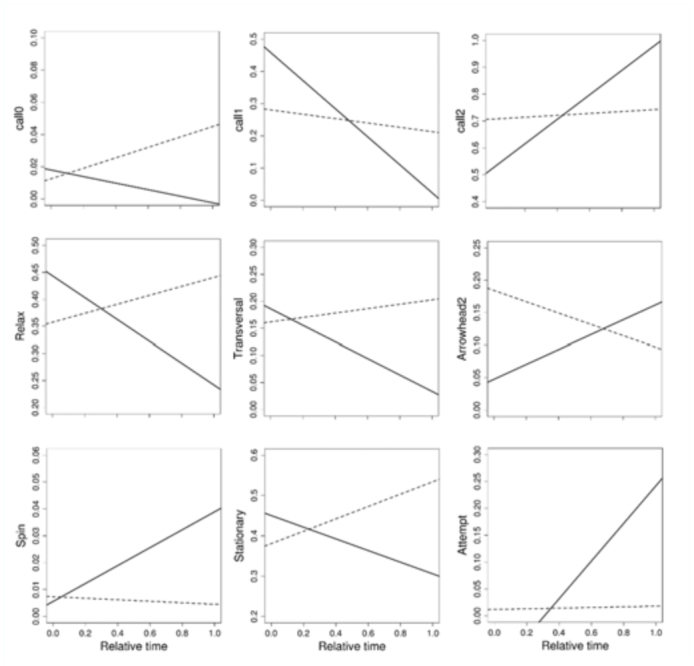
Regression plots representing the time spent on each activity (described in Table 1) as a function of the relative time taken between female release and copulation or the end of the observation period. Only the cases where the interaction component in Table 3 was significant are illustrated. Solid line = Successful individuals (S). Dotted line = Unsuccessful individuals (U). High quality figures are available online.

As total courtship time differed between individuals (from 14 seconds to 30 minutes) the tests were conducted with relative times. The ANCOVA of the time spent on each activity along courtship time (see Table 3) showed that in 10 out of 16 behavioral traits considered the regression was significant or highly significant (Table 3, column 4). In all cases where the interaction (Table 3, column 5) between success and relative time was non- significant (that is, the plots for S and U males were parallel), the slope was negative. Within this group, the intercept differed significantly between S and U males for Arrowhead 1 and Fanning (Table 3, column 3). In both cases, U males showed a lower intercept. The interaction was significant or highly significant for nine traits (Table 3, column 5). These results indicate that success might be attributed to the differences in the investment in different activities. To show the results more clearly, the difference in trends between S and U individuals for those traits where the interaction was significant is graphically presented (Figure 1). Clearly, as courtship proceeds S males tend to increase (positive slope) the time dedicated to Call 2, Arrowhead 2, Spin, and Attempt and decrease (negative slope) the time allocated to the remaining activities. The trends are clearly different for U individuals, which do not show an increase of the time dedicated to activities directly oriented to mating such as Arrowhead 2, Spin, and Attempt.

**Table 4.**
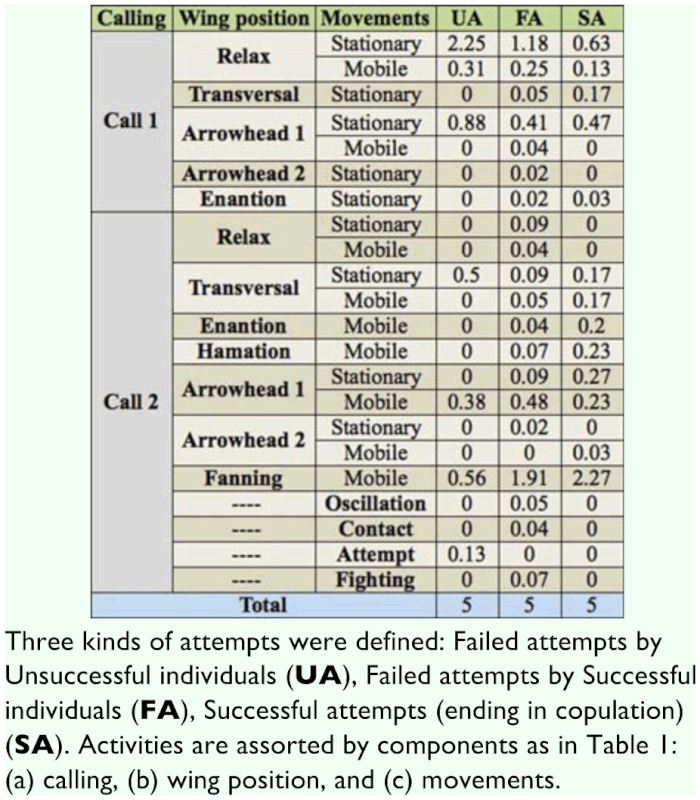
Average time (in seconds) dedicated by the males to each activity during the five seconds before each copulation attempt.

**Table 5.**
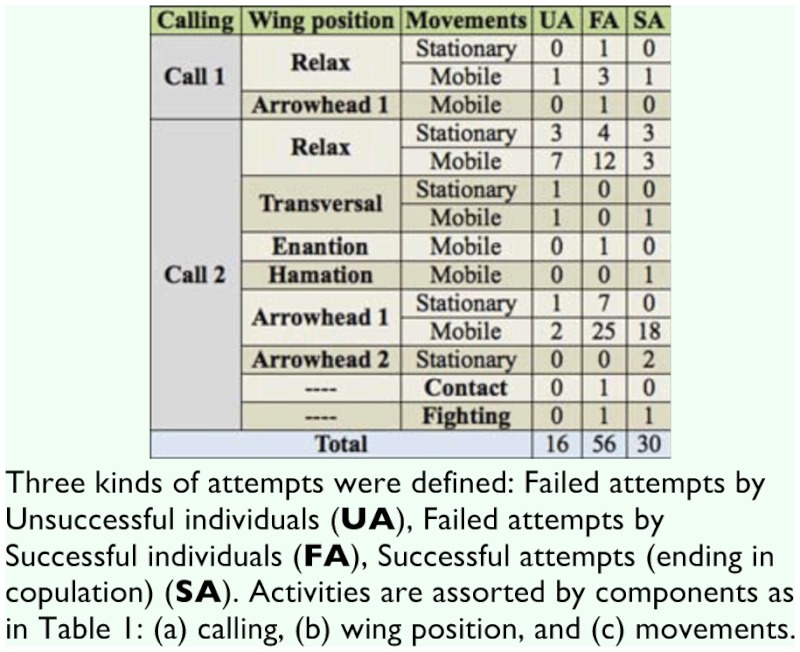
Frequency of the last activity of the males before each copulation attempt.

**Figure 2.**
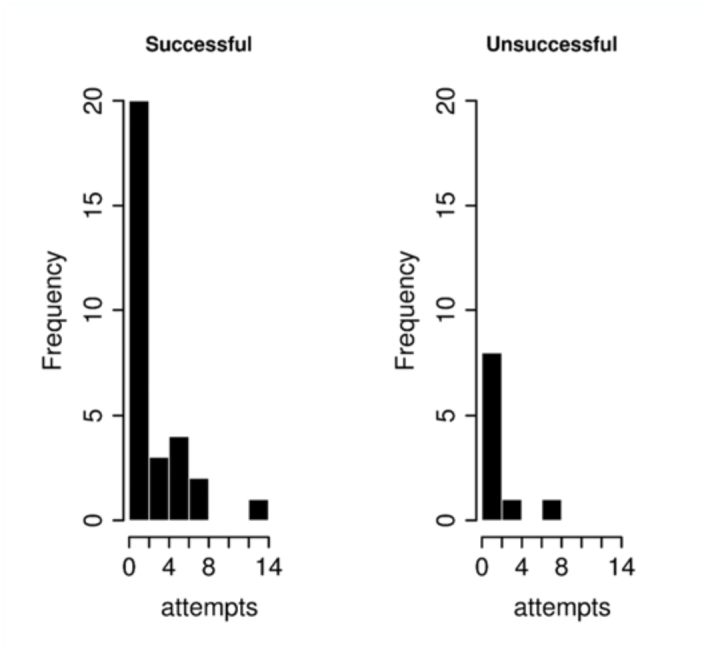
Histograms representing the absolute frequency of different numbers of mating attempts per individual for Successful and Unsuccessful males. High quality figures are available online.

During the video recordings, S males made a total of 87 Attempts, 31 of which finished in copulation (successful Attempts, SA), and 56 were rejected (failed Attempts, FA). Among U males, five individuals did not make any Attempt during the recording time. The remaining U males made a total of 16 failed Attempts (UA). The frequency distribution of number of Attempts is given in Figure 2 for S and U males. The average number of Attempts (2.9 and 1.6 for S and U respectively) differs significantly between these groups (z = –2.142,*p* < 0.05)

**Figure 3.**
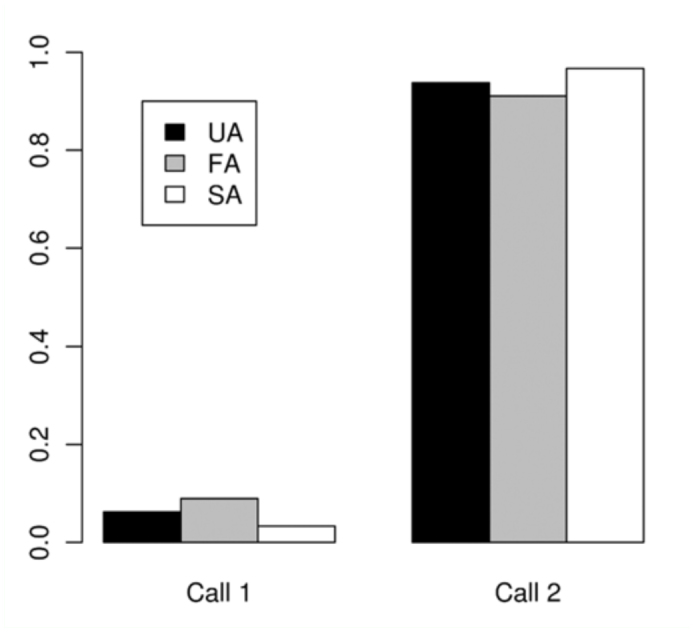
Average time dedicated to each calling activity (described in Table 1) by males during the five seconds previous to the copulation attempts. Failed attempts by Unsuccessful individuals (**UA**), Failed attempts by Successful individuals (**FA**), Successful attempts (ending in copulation) (**SA**). High quality figures are available online.

**Figure 4.**
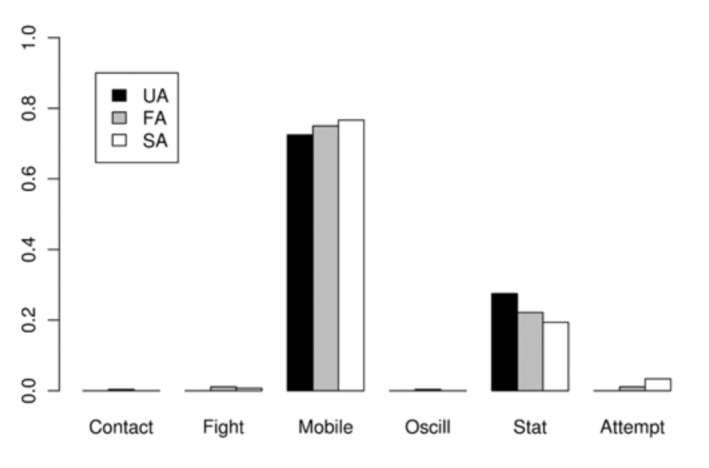
Average time dedicated to each movement activity (described in Table 1) by males during the five seconds previous to the copulation attempts. Failed attempts by Unsuccessful individuals (**UA**), Failed attempts by Successful individuals (**FA**), Successful attempts (ending in copulation) (**SA**). Abbreviations: **Fight,** Fighting; **Oscill,** Oscillation; **Stat**, Stationary. High quality figures are available online.

**Figure 5.**
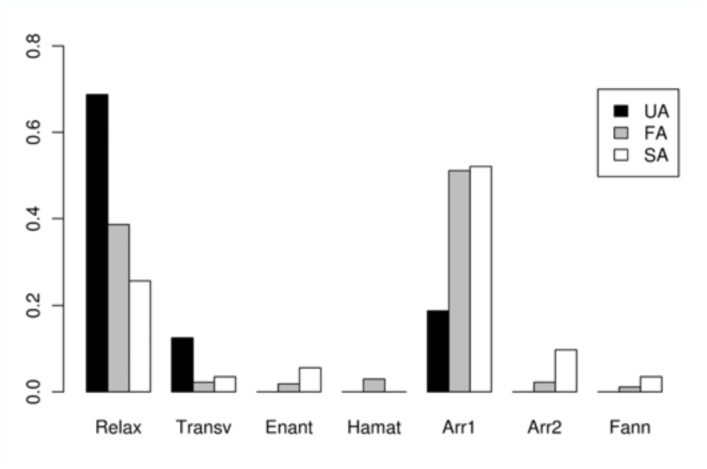
Average time dedicated to each wing position activity (described in Table 1) by males during the five seconds previous to the copulation attempts. Failed attempts by Unsuccessful individuals (**UA**), Failed attempts by Successful individuals (**FA**), Successful attempts (ending in copulation) (**SA**). Abbreviations: **Transv**, Transversal; **Enant**, Enantion; **Hamat**, Hamation; **Arr**, Arrowhead; **Fann**, Fanning. High quality figures are available online.

The time dedicated to each activity during the five seconds before different Attempt classes (SA, FA, and UA) is shown in Table 4. The proportion of time spent on each activity before each of these Attempts is shown in Figures 3–5 assorted by components. No statistical differences were detected for calling (χ^2^ = 4.84, > 0.05) or movement (χ2 = 8.52, *p* > 0.05) activities, among the three classes. For wing activities, highly significant differences were found when all three classes were compared (χ^2^ = 95.59, *p* < 0.01), and all pairwise comparisons were also highly significant (χ^2^ = 27.70–62.69, *p* < 0.01). Clearly, U individuals remain in a more passive position, with their wings in Relax or Transversal, while S individuals exhibited more elaborate displays such as Arrowhead, Hamation and Enantion. SAs were usually preceded by a longer time in Arrowhead 1 or 2, and a shorter time in Relax than UA and FA.

**Figure 6.**
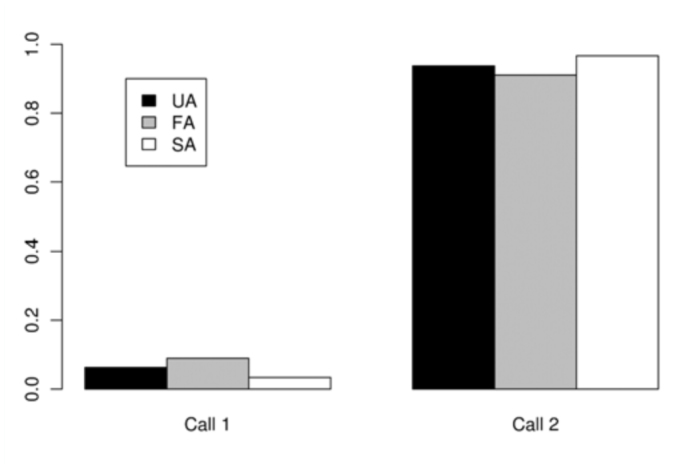
Average frequency of occurrence of each calling activity (described in Table 1) immediately before the copulation attempts. Failed attempts by Unsuccessful individuals (**UA**), Failed attempts by Successful individuals (**FA**), Successful attempts (ending in copulation) (**SA**). High quality figures are available online.

**Figure 7.**
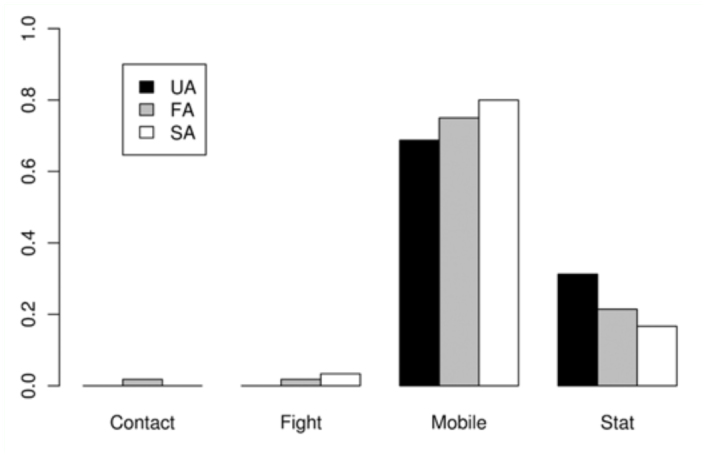
Average frequency of occurrence of each movement activity (described in Table 1) immediately before the copulation attempts. Failed attempts by Unsuccessful individuals (**UA**), Failed attempts by Successful individuals (**FA**), Successful attempts (ending in copulation) (**SA**). Abbreviations: **Fight**, Fighting; **Stat**, Stationary. High quality figures are available online.

**Figure 8.**
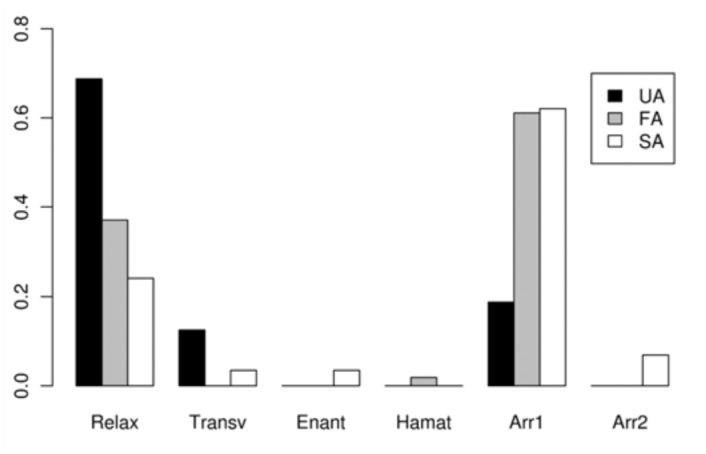
Average frequency of occurrence of each wing position activity (described in Table 1) immediately before the copulation attempts. Failed attempts by Unsuccessful individuals (**UA**), Failed attempts by Successful individuals (**FA**), Successful attempts (ending in copulation) (**SA**). Abbreviations: **Transv**, Transversal; **Enant**, Enantion; **Hamat**, Hamation; **Arr**, Arrowhead. High quality figures are available online.

The absolute frequency of the occurrence of each activity as the last one immediately before the Attempts was registered (Table 5) and compared among the three classes of Attempts. 21 out of 31 successful males showed Arrowhead (in most cases: Arrowhead 1 with Mobile) just before the Attempt that finished in copulation (SA). The time spent on that position was variable (1–25 seconds). No significant differences were detected among the three classes of Attempts for calling (*p* = 0.7731) (Figure 6) or movement (*p =* 0.5697). The latter comparison was based only on Mobile and Stationary due to the low frequency of Contact and Fight (Figure 7). In general, S individuals exhibited more wing displays than U individuals. Significant differences were found (*p* < 0.01) when wing positions were compared among SA, FA, and UA. In this case, comparisons were based on Relax and Arrowhead 1—the most frequent activities (Figure 8). Pairwise comparisons showed significant differences between FA and UA (*p *< 0.01), but not between SA and FA (*p* = 0.4533).

Matings lasted from 19 to 125 minutes, with a mean of 63 — 24 min SD. Results of quantitative observations during the copulation itself were not included, but couples clearly spent most of the time in the same spot without major displacements, except for occasional body rearrangements when the male repositioned himself over the female. The male often touched the female's head with his proboscis, which appeared to have a calming action over her.

### Discussion

The current study was conducted during the period of maximum mating activity observed under field cage experiments (Allinghi et al. 2007). Males began calling on the rearing flask and continued doing so inside the cage where recordings were conducted. The males spent almost 90% of the time on releasing pheromone, which probably is an accurate representation of what would happen in the wild from dawn until 10:00 or 11:00 in the morning.

Almost 50% of matings occurred within five minutes of female introduction and the quickest copulation happened only 14 seconds after the female placement. Such short latency and courtship time could be related with rearing conditions. For example, in other tephritid species such as *C. capitata*, it has been shown that mass rearing conditions favor a reduction in courtship duration, an increase female acceptance, and a reduction in copulation duration (Calcagno et al. 1999; Briceño and Eberhard 2000). However, the average copulation duration (63 minutes) recorded in the present trial is similar to other measurements obtained by our group when wild *A. fraterculus* were tested in field cages; 65 to 67.30 min according to Petit-Marty et al. (2004) and Segura et al. (2007), respectively.

There is also evidence suggesting that the mating behavior of the laboratory strain studied here is similar to that of wild flies, as Allinghi et al. (2007) demonstrated high mating compatibility under field cage conditions between this strain and wild collected flies.

There was great variation among males in the display of individual activities, which is consistent with courtship observations in other tephritids, such as *C. capitata* (Briceño et al. 1996; Liimatainen et al. 1997; Briceño and Eberhard 2002). There was not a precise or unique sequence leading to mating; however, some pronounced differences were observed between successful and unsuccessful males. Notably, S males generally spent relatively more time performing Arrowhead 2 and Spin than did the U males through time. U males changed their behavior through time to activities not directly aimed to female attraction, remaining quieter (Stationary) and with their wings in a passive position (Relax or Transversal). Call 2 appeared to be a requisite for reaching copulation (only one successful Attempt was not preceded by Call 2). With respect to wing positions, in successful individuals, ∼ 67% of successful Attempts and 59% of failed Attempts were preceded by Arrowhead 1 or Arrowhead 2. This contrasts with observations in unsuccessful individuals where only 19% of Attempts were preceded by these positions. Spin appeared to influence copulatory success because this activity was displayed only in female presence. The relative time spent in Spin by unsuccessful males was much shorter than by successful males, and this activity increased as courtship progressed. However, this behavior was not observed within the five seconds before the Attempts, suggesting that a shift to Arrowhead is necessary before a mating Attempt. The apparent need of a combination of activities (Call 2, Spin, Arrowhead) to increase mating success supports the hypothesis that female acceptance is influenced by a large number of factors that slightly improve the chance of mating success rather than guaranteeing it (Briceño and Eberhard 2002).

Although Call 2, Spin, and Arrowhead seem to be important components of mating success, the sequence of activities showed variation within successful individuals. Hence, there is a difference from results obtained with similar experimental conditions for *C. capitata* (Calcagno et al. 1999), where courtship mainly exhibited the same steps, and the copulation Attempt was normally preceded by sudden wing agitations with a piercing noise (Buzzing). Another difference between *C. capitata* and *A. fraterculus *courtships is that wing vibration is not so important in the latter, where this energetically expensive display did not always lead to copulation, and no significant differences were found between successful and unsuccessful individuals under the current experimental conditions. Nevertheless, it is necessary to consider the possibility that this display is more useful in the wild by making the male more apparent to approaching females or by attracting other males to form a lek.

The number of Attempts per individual was variable; five males never intended copulation, and one male achieved copulation only in the 13^th^ Attempt. The average number of Attempts per individual was lower in U than in S individuals, which could represent different motivation among the males or a response to the behavior of the female. However, some insight may be attained from the comparison of (1) the behavior of U and S males before failed Attempts and (2) failed and successful Attempts by S males. In the first case the female was reluctant and the male was rejected, but male behavior after rejection was different between S and U males. Rejected U males increased the time spent on Relax at the expense of Arrowhead 1, and attempted mounting (Attempt) less times than rejected S males. By contrast, the second comparison indicates no significant differences in behavior among S no matter the female response. Rejected S males remained dedicated to activities directly related with mating success (Arrowhead 1 and Arrowhead 2) and attempted to mount (Attempt) until female acceptance. These results support the hypothesis that male behavior is at least partially responsible for final female acceptance, rather than a simple consequence of female predisposition.

The conclusions attained in this work should be compared with analyses based on wild individuals. However, major differences would not be expected, since compatibility and competitiveness tests conducted under field cage conditions did not show isolation between this strain of laboratory reared flies and counterparts emerged from field collected fruits (Allinghi et al. 2007). Periodical tests conducted on laboratory strains would allow evaluation of possible departures from the wild normal behavior, especially with respect to the number of successful Attempts and the wing position behavior.

## **Abbreviations: **

S: successful;
U: unsuccessful;
UA: failed attempts by unsuccessful males;
FA: failed attempts by successful males;
SA: successful attempts (ending in copulation)>;
